# Diphlorethohydroxycarmalol from *Ishige okamurae* Suppresses Osteoclast Differentiation by Downregulating the NF-κB Signaling Pathway

**DOI:** 10.3390/ijms18122635

**Published:** 2017-12-06

**Authors:** Hye Jung Ihn, Ju Ang Kim, Hye Sung Cho, Hong-In Shin, Gi-Young Kim, Yung Hyun Choi, You-Jin Jeon, Eui Kyun Park

**Affiliations:** 1 Department of Oral Pathology and Regenerative Medicine, School of Dentistry, Institute for Hard Tissue and Biotooth Regeneration, Kyungpook National University, Daegu 41940, Korea; hjpihn@hanmail.net (H.J.I.); kkangjin5@hanmail.net (J.A.K.); uricomet@knu.ac.kr (H.S.C.); hishin@knu.ac.kr (H.-I.S.); 2 Department of Marine Life Science, Jeju National University, Jeju 63243, Korea; immunkim@jejunu.ac.kr (G.-Y.K.); youjinj@jejunu.ac.kr (Y.-J.J.); 3 Department of Biochemistry, College of Oriental Medicine, Dong-Eui University, Busan 47227, Korea; choiyh@deu.ac.kr

**Keywords:** diphlorethohydroxycarmalol, brown algae, osteoclast, NF-κB

## Abstract

Marine algae possess a variety of beneficial effects on human health. In this study, we investigated whether diphlorethohydroxycarmalol (DPHC), isolated from *Ishige okamurae*, a brown alga, suppresses receptor activator of nuclear factor-κB ligand (RANKL)-induced osteoclast differentiation. DPHC significantly suppressed RANKL-induced osteoclast differentiation and macrophage-colony stimulating factor (M-CSF) expression in a dose-dependent manner. In addition, it significantly inhibited actin ring formation, the expression of osteoclast marker genes, such as tartrate-resistant acid phosphatase (TRAP), nuclear factor of activated T-cells cytoplasmic 1 (Nfatc1), cathepsin K (Ctsk), and dendritic cell-specific transmembrane protein (Dcstamp), and osteoclast-induced bone resorption. Analysis of the RANKL-mediated signaling pathway showed that the phosphorylation of both IκB and p65 was specifically inhibited by DPHC. These results suggest that DPHC substantially suppresses osteoclastogenesis by downregulating the RANK-NF-κB signaling pathway. Thus, it holds significant potential for the treatment of skeletal diseases associated with an enhanced osteoclast activity.

## 1. Introduction

Osteoclasts are a type of specialized multinucleated cells (MNCs) that cause bone resorption, whereas osteoblasts cause bone formation; these cells are active throughout our lifetime. Bone formation and bone resorption are functionally coupled and tightly regulated to maintain normal bone remodeling. However, the abnormal activation and formation of osteoclasts are responsible for skeletal diseases, such as osteoporotic, metastatic, and inflammatory bone loss [1,2]. Menopause, aging, inflammation, and cancer metastasis can lead to enhanced osteoclast activation [3,4]. Therefore, it is necessary to reduce osteoclast differentiation and/or its bone-resorption effects to manage such bone diseases.

Receptor activator of nuclear factor-κB ligand (RANKL) and macrophage colony-stimulating factor (M-CSF) are expressed by osteoblasts/stromal cells, and play key roles in the differentiation of osteoclast precursors and the bone-resorbing activity of osteoclasts [5,6]. Activation of RANK by RANKL induces mitogen-activated protein kinase and nuclear factor-κB (NF-κB) signaling. These signaling pathways lead to an increase in the expression of osteoclast-specific genes and bone resorption [7,8].

Natural resources contain pharmacologically active components; marine algae are used as a source of functional food [9]. They contain diverse bioactive compounds with great pharmaceutical and biomedical potential. In particular, brown algae are composed of a variety of biological compounds, including pigments, fucoidans, phycocolloids, and phlorotannins, and exert antioxidant, anticoagulant, antihypertensive, antibacterial, antitumor, antidiabetic, and anti-inflammatory activities [10,11,12,13]. Diphlorethohydroxycarmalol (DPHC) is a type of phlorotannin isolated from the brown alga, *Ishige okamurae*, which is abundantly found along the coast of Jeju Island in Korea. DPHC is reported to alleviate postprandial hyperglycemia in diabetic mice and hyperglycemia-induced oxidative stress in human umbilical vein endothelial cells [10,14]. In addition, DPHC reduces the production of interleukin 6, but not of tumor necrosis factor-α induced by lipopolysaccharide in murine macrophages [11]. However, its anti-osteoclastic activity remains unexplored.

In this study, we isolated and evaluated the effects of DPHC on osteoclast differentiation and bone resorption, and found that DPHC suppresses both osteoclast differentiation and its functions in vitro.

## 2. Results

### 2.1. Effects of Diphlorethohydroxycarmalol (DPHC) on the Viability of Bone Marrow Macrophages (BMMs) and Osteoclast Formation

**Figure 1:**
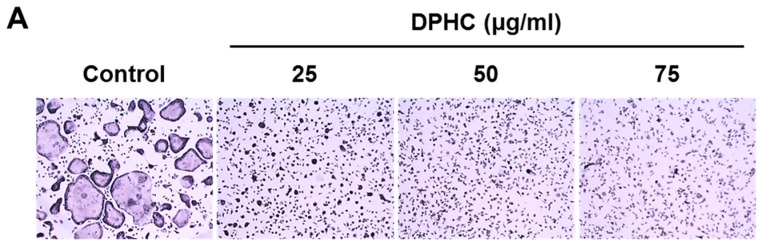

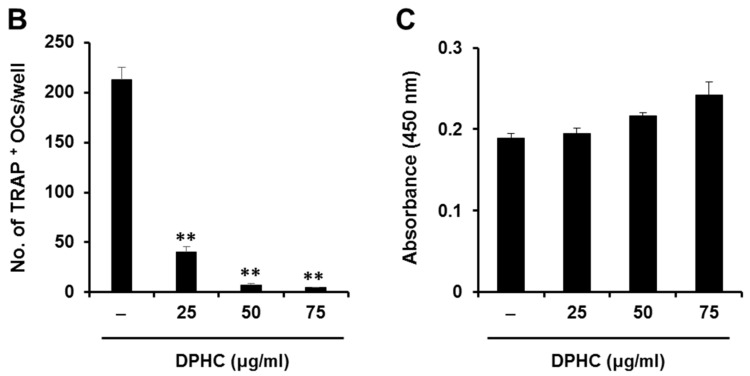
Effects of diphlorethohydroxycarmalol (DPHC) on receptor activator of nuclear factor-κB ligand (RANKL)-mediated osteoclast differentiation in bone marrow macrophages (BMMs). (**A**) BMMs were cultured for four or five days in osteoclastogenic media containing macrophage-colony stimulating factor (M-CSF) (10 ng/mL) and RANKL (20 ng/mL) with or without various doses of DPHC. The cells were stained for TRAP; (**B**) The number of TRAP-positive multinucleated cells (MNCs) with ≥3 nuclei was scored. ** *p* < 0.01 versus vehicle-treated control; (**C**) BMMs were incubated with the indicated doses of DPHC in the presence of M-CSF (10 ng/mL) for three days. Cell viability was assessed by the cell counting kit-8 (CCK-8). TRAP: tartrate-resistant acid phosphatase; OCs: osteoclasts.

To examine whether DPHC inhibits RANKL-induced osteoclast formation from bone marrow macrophages (BMMs), mouse BMMs were treated with M-CSF (10 ng/mL) and RANKL (20 ng/mL) in the absence or presence of 25, 50, or 75 μg/mL DPHC. M-CSF and RANKL (positive controls) increased the number of tartrate-resistant acid phosphatase (TRAP)-positive MNCs, whereas treatment with DPHC substantially reduced this number and inhibited osteoclast differentiation (Figure 1A). Osteoclast formation stimulated by M-CSF and RANKL was inhibited by DPHC in a dose-dependent manner (Figure 1B). At 75 μg/mL, it reduced the number of TRAP-positive MNCs by 97% compared to control (*p* < 0.01) (Figure 1C). To determine the specificity of this inhibitory effect of DPHC on osteoclast differentiation, cytotoxicity of DPHC was analyzed with the cell counting kit-8 (CCK-8). BMMs were treated with M-CSF in the presence or absence of DPHC for three days. The results showed that DPHC did not affect the viability of BMMs at the concentration of 75 μg/mL (Figure 1C).

**Figure 2:**
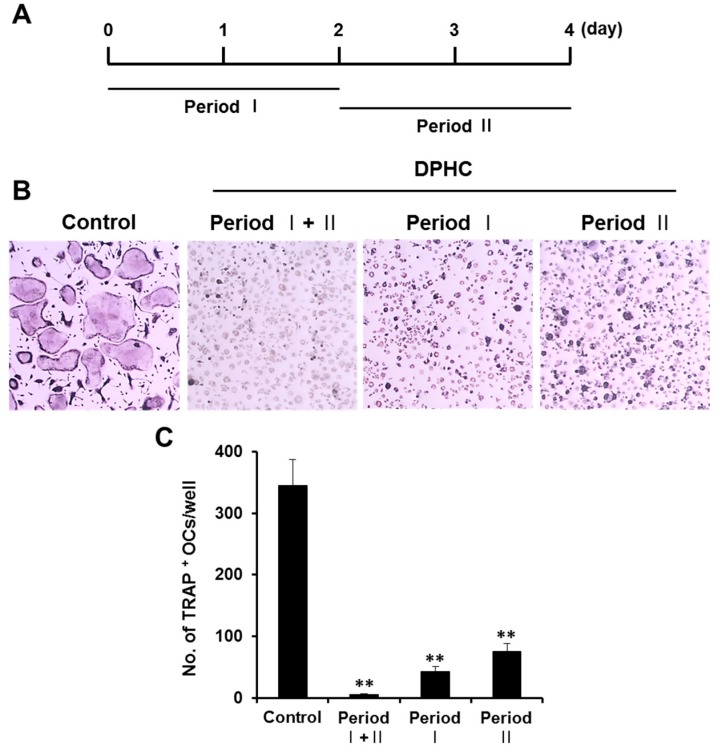
Effects of DPHC on osteoclast formation after exposing BMMs for different time periods to DPHC. (**A**) Schematic representation of the in vitro experiment; (**B**) BMMs were cultured in osteoclastogenic media with DPHC (75 μg/mL) added during different stages of cell culture. Osteoclast formation was confirmed by TRAP staining; (**C**) TRAP-positive multinucleated cells (MNCs) were counted; ** *p* < 0.01 versus vehicle-treated control.

We further analyzed the stage-specific effect of DPHC on osteoclast differentiation. In our system, pre-osteoclasts are generated from BMMs after two days of incubation with RANKL (period I), and they further differentiate into mature osteoclasts by RANKL (period II). Exposure to DPHC from culture initiation (seeding) to the end of culture (period I + II) abolished osteoclast formation (Figure 2A,B). TRAP-positive MNCs were rarely observed upon DPHC exposure from seeding till pre-osteoclast formation (period I), and few TRAP-positive MNCs were observed upon DPHC exposure after pre-osteoclast formation (period II) (Figure 2B). Overall, the number of TRAP-positive MNCs decreased by 78% upon DPHC exposure (Figure 2C), suggesting that it suppresses all stages of osteoclast differentiation, and more efficiently the formation of pre-osteoclasts from BMMs.

### 2.2. Effects of DPHC on Actin Ring Formation and NFATc1 Expression/Localization

**Figure 3:**
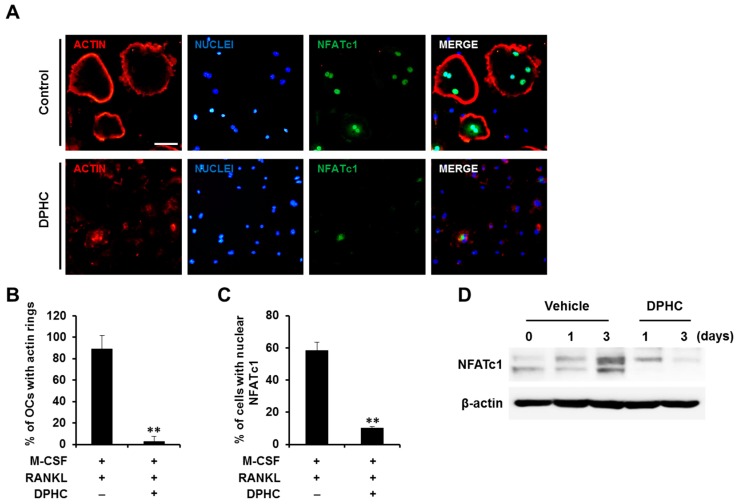
Effects of DPHC on actin ring formation and NFATc1 expression. (**A**) BMMs were cultured on glass coverslips in osteoclastogenic media with vehicle or DPHC (75 μg/mL) for four days. Further, the cells were labeled with anti-NFATc1 antibody, followed by phalloidin and 4′,6-diamidino-2-phenylindole (DAPI) staining; scale bar, 50 μm; (**B**) The number of actin rings per bone slice and (**C**) the number of cells with nuclear NFATc1 were counted; ** *p* < 0.01 versus vehicle-treated control; (**D**) BMMs were cultured in osteoclastogenic media in the absence or presence of DPHC (75 μg/mL) for the indicated durations. NFATc1 protein levels in the cell lysates were analyzed by western blotting.

Osteoclasts are morphologically characterized by actin ring formation [15]. Therefore, we evaluated the effect of DPHC on this morphological development in MNCs. Rearrangement of actin cytoskeleton and subsequent actin ring formation were induced by M-CSF and RANKL (Figure 3A). However, DPHC significantly inhibited the formation of actin rings (Figure 3A,B). 4′,6-diamidino-2-phenylindole (DAPI) staining showed that the nuclei were intact (Figure 3A), and immunostaining analysis showed that DPHC significantly reduced the nuclear localization of NFATc1 (Figure 3A,C). In addition, the protein level of NFATc1 was also markedly decreased by DPHC (Figure 3D).

### 2.3. Effect of DPHC on the Expression of Osteoclastic Markers

**Figure 4:**
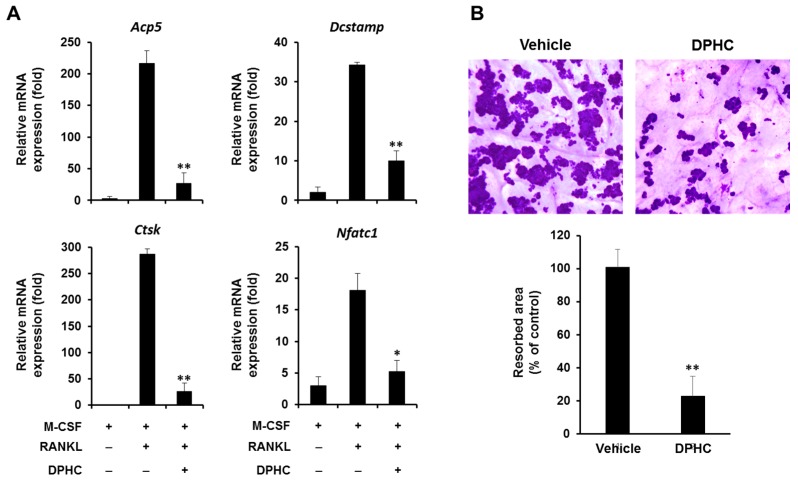
Effects of DPHC on RANKL-induced gene expression and the formation of resorption pits. (**A**) BMMs were cultured with M-CSF (10 ng/mL) and RANKL (20 ng/mL) for four days in the absence or presence of DPHC (75 μg/mL). The mRNA expression of TRAP (*Acp5*), DC-STAMP (*Dcstamp*), Cathepsin K (*Ctsk*), and NFATc1 (*Nfatc1*) was analyzed using real-time PCR; (**B**) Mouse BMMs were seeded on bone slices and cultured with M-CSF (10 ng/mL) and RANKL (20 ng/mL) for three days to induce osteoclast differentiation from BMMs. Further, the cells were treated with or without DPHC (75 μg/mL) for two days. The resorption pits were stained with hematoxylin solution, and stained areas were measured; * *p* < 0.05; ** *p* < 0.01 versus vehicle-treated control.

To further confirm the inhibitory effect of DPHC on osteoclast differentiation, we evaluated the mRNA expression of osteoclastic marker genes. Consistent with the decreased protein level and nuclear translocation of NFATc1, DPHC suppressed the expression of osteoclast-specific genes, including *Acp5* and *Ctsk* (Figure 4A). *Dcstamp* encodes for the dendritic cell-specific transmembrane protein, Dcstamp, a chief regulator of cell fusion in pre-osteoclasts. Along with reduced cell fusion and actin ring formation, *Dcstamp* expression was also downregulated by DPHC (Figure 4A).

### 2.4. Effect of DPHC on Bone Resorption

Because DPHC significantly inhibited osteoclast differentiation, we evaluated whether it affects the bone resorbing activity of mature osteoclasts. To test osteoclast activity, we cultured osteoclast-like MNCs from BMMs on bone slices in the presence of M-CSF and RANKL. Further, the cells were incubated with or without DPHC for two days in osteoclastogenic media, and the slices were stained with hematoxylin solution. Treatment with DPHC reduced the formation of resorption pits by 77% compared to control (Figure 4B). These results indicate that DPHC significantly inhibits osteoclast-induced bone resorption and osteoclast differentiation.

### 2.5. Effect of DPHC on the RANKL-Induced Signaling Pathway

**Figure 5:**
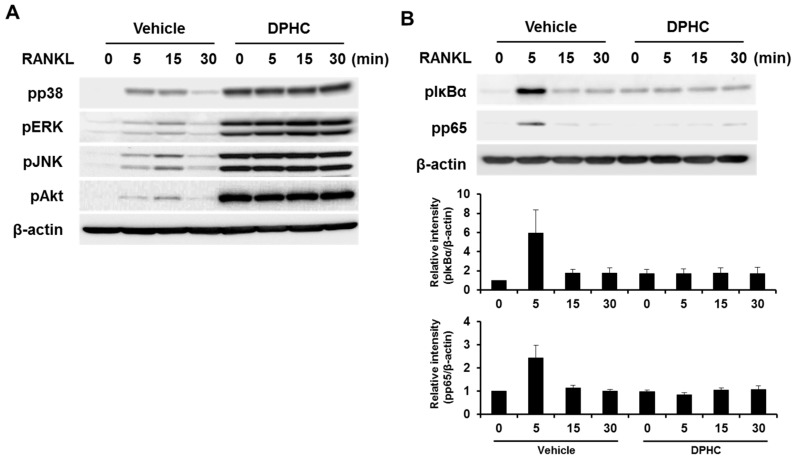
Effects of DPHC on the RANKL-induced signaling pathway. (**A**,**B**) Mouse BMMs were incubated in serum-free medium for five hours and pretreated with vehicle or DPHC (75 μg/mL) for one hour. The cells were stimulated with RANKL (50 ng/mL) for the indicated durations, and immunoblotting was performed using specific antibodies against phosphorylated forms of p38, ERK, JNK, Akt, IκB, and p65; β-actin was used as the loading control.

To understand the mechanisms underlying the inhibitory effects of DPHC, BMMs were pretreated with DPHC or vehicle, stimulated with RANKL for various durations, and the phosphorylation of downstream signaling molecules was analyzed by western blotting. RANKL stimulation induced the phosphorylation of p38, ERK, JNK, and Akt, and DPHC further induced their phosphorylation (Figure 5A). However, the phosphorylation of IκB and p65 was significantly inhibited by DPHC (Figure 5B), indicating that it inhibits RANKL-induced osteoclastogenesis and osteoclast-induced bone resorption by attenuating the activation of the NF-κB signaling pathway.

## 3. Discussion

The balance between osteoblast-induced bone formation and osteoclast-induced bone resorption is critical for bone homeostasis. An imbalance, mostly caused by enhanced bone resorption due to an increase in osteoclast activation, leads to skeletal diseases. Bisphosphonates (BPs) are commonly prescribed for the treatment of skeletal diseases. They potently inhibit osteoclast formation and survival [16,17]. Because osteoblast–osteoclast activities are tightly regulated by various growth factors [18,19], an inordinate inhibition of osteoclast formation and function may impair osteoblast–osteoclast coupling and bone remodeling processes. This could explain the development of osteonecrosis in the jaw as an adverse effect of BPs. Recently developed monoclonal antibodies, such as denosumab, targeting RANK on the surface of osteoclasts, may also cause similar adverse effects [20]. Therefore, natural compounds are being explored for the maintenance of normal osteoblast–osteoclast coupling and bone remodeling, and to avoid the adverse effects of anti-osteoclastic BPs. Various herbal medicines have been reported to improve bone health [21]. In this study, we demonstrate that DPHC, a major component of marine brown algae, inhibits osteoclast differentiation and function. DPHC significantly inhibited the formation of MNCs with no significant cytotoxicity (Figure 1). Moreover, DPHC inhibited both early (commitment to osteoclast and pre-osteoclast formation) and late (multinucleation and activation of mature osteoclasts) phases of osteoclastogenesis; the number of osteoclasts was similarly reduced regardless of the time at which the cells were exposed to DPHC (Figure 2). Moreover, DPHC almost completely inhibited actin ring formation, which is observed in MNCs (Figure 3A), suggesting that DPHC may suppress osteoclast maturation and function.

In addition to suppressing the morphological development of osteoclasts, DPHC significantly inhibited the expression of osteoclastic marker genes (Figure 4A). These results strongly suggest that DPHC inhibits osteoclast differentiation. This was further supported by the significantly reduced expression of *Nfatc1*, the primary regulator of osteoclast differentiation, upon DPHC exposure (Figure 4A). Therefore, we hypothesized that DPHC targets upstream signaling pathways leading to the reduced expression of *Nfatc1*, which regulates the expression of osteoclast differentiation markers, such as TRAP, cathepsin K, calcitonin receptors, and osteoclast-associated receptors, by interacting with MITF and c-Fos proteins [22,23,24,25].

*Nfatc1* expression during osteoclast differentiation is regulated by several transcription factors, including c-Fos and NF-κB [26,27,28]. c-Fos knockout mice exhibit osteopetrosis due to defects in committing to osteoclast differentiation [26,28]. Dehydroxymethylepoxyquinomicin, a pharmacological inhibitor of NF-κB, attenuates RANKL-induced osteoclast differentiation by downregulating *Nfatc1* expression [27]. We found that DPHC significantly inhibited RANKL-induced NF-κB activation, which was observed by the phosphorylation of IκB and p65 (Figure 5B). These results strongly suggest that DPHC targets the NF-κB signaling pathway, and downregulates the expression of *Nfatc1*. DPHC has been shown to inhibit lipopolysaccharide-induced NF-κB activation during macrophage activation [11], further supporting our observation that DPHC targets the NF-κB pathway. Interestingly, phosphorylation of p38, ERK, JNK, and Akt was increased by DPHC (Figure 5A). Although the underlying molecular mechanisms are not clear, the specific inhibition of p38, ERK, JNK, and Akt failed to prevent DPHC-mediated inhibition of osteoclast differentiation. These results suggest that the integrity of the signaling pathways activated by RANKL, including p38, ERK1/2, JNK, AKT and NF-κB pathways is critical for proper osteoclast differentiation. In summary, we found that DPHC, extracted from marine brown algae, significantly suppresses the differentiation as well as the bone resorbing activity of osteoclasts by downregulating the NF-κB signaling pathway.

## 4. Materials and Methods

All animal experiments were approved by the Committee on the Care and Use of Animals in Research at Kyungpook National University (KNU 2017-74), and were conducted in accordance with the guidelines for the care and use of laboratory animals.

### 4.1. Antibodies and Reagents

Antibodies against phospho-p38, phospho-ERK, phospho-JNK, phospho-Akt, phospho-IκBα, and phospho-p65 were purchased from Cell Signaling Technology (Danvers, MA, USA). Monoclonal anti-β-actin antibody was obtained from Sigma-Aldrich (St. Louis, MO, USA), and anti-Nfatc1 antibody was purchased from BD Pharmingen (San Diego, CA, USA). Recombinant M-CSF and RANKL were purchased from R & D Systems (Minneapolis, MN, USA). DPHC was isolated from *Ishige okamurae*, and the method of isolation as well as the purity and structure of DPHC were confirmed by spectroscopy [9].

### 4.2. Osteoclast Differentiation

Osteoclast differentiation was induced as previously described [29,30]. The bone marrow of long bones from 6–8-week-old male C57/B6L mice was isolated, and the cells were cultured in α-minimum essential medium (α-MEM) containing 10% fetal bovine serum (FBS). On the next day, floating cells were harvested and further cultured with M-CSF (30 ng/mL) in α-MEM supplemented with 10% FBS for three days to obtain BMMs. To evaluate the effect of DPHC on osteoclastogenesis, BMMs were cultured in 96-well plates with RANKL (20 ng/mL) and M-CSF (10 ng/mL) in the absence or presence of various concentrations of DPHC. After four days, the cells were fixed in 4% paraformaldehyde and stained for TRAP activity using an acid phosphatase leukocyte staining kit (Sigma-Aldrich). TRAP-positive MNCs containing three or more nuclei were considered as osteoclast-like cells.

### 4.3. Cell Viability Assay

The cytotoxic effect of DPHC was determined using CCK-8 (Dojindo Molecular Technologies Inc., Rockville, MD, USA). BMMs were cultured in α-MEM containing 10% FBS and M-CSF (10 ng/mL) with or without DPHC for three days. Further, the cells were incubated with α-MEM containing 10% CCK-8 reagent at 37 °C for 2 h. The absorbance was measured at 450 nm using a microplate reader (BioRad, Hercules, CA, USA).

### 4.4. RNA Isolation and Real-Time PCR

To analyze the mRNA expression of osteoclastogenic factors, BMMs were cultured with DPHC (75 μg/mL) or vehicle for four days. Total RNA was isolated with the TRI solution (Bioscience, Seoul, Korea) and used for cDNA synthesis. Real-time PCR was conducted on a LightCycler 1.5 real-time PCR system (Roche Diagnostics, Basel, Switzerland) using the SYBR Premix Ex Taq (Takara Bio Inc., Shiga, Japan). The following mouse primer sequences were used: *Acp5* or TRAP, 5′-TCCCCAATGCCCCATTC-3′ and 5′-CGGTTCTGGCGATCTCTTTG-3′; *Ctsk*, 5′-GGCTGTGGAGGCGGCTAT-3′ and 5′-AGAGTCAATGCCTCCGTTCTG-3′; *Dcstamp*, 5′-CTTCCGTGGGCCAGAAGTT-3′ and 5′-AGGCCAGTGCTGACTAGGATGA-3′; *Nfatc1*, 5′-ACCACCTTTCCGCAACCA-3′ and 5′-TTCCGTTTCCCGTTGCA-3′.

### 4.5. Western Blotting

Total protein was isolated from the cultured cells using a RIPA lysis buffer containing protease and phosphatase inhibitors. Protein concentration was evaluated using a bicinchoninic acid (BCA) assay kit (Pierce Biotechnology, Rockford, IL, USA), and equal amounts of protein (25 μg) were loaded and separated using 10% sodium dodecyl sulfate-polyacrylamide gel electrophoresis (SDS-PAGE). The separated proteins were transferred onto nitrocellulose membranes (Whatman, Florham Park, NJ, USA), blocked with 3% non-fat dry milk in Tris buffered saline-Tween 20 (TBST) (25 mM Tris-HCl, pH 7.4, 150 mM NaCl, and 0.2% Tween 20), and then incubated with primary antibodies (1:1000) at 4 °C overnight. The membranes were washed and incubated with secondary antibodies for 1 h. Immunoreactivity was detected using WesternBright ECL (Advansta, Menlo Park, CA, USA) and a chemiluminescence imager (Azure Biosystems, Inc., Dublin, CA, USA).

### 4.6. Immunofluorescence

After seeding on glass coverslips, BMMs were cultured in osteoclastogenic media with DPHC (75 μg/mL) or vehicle. After four days, the cells were fixed with 4% paraformaldehyde and permeabilized with 0.25% Triton X-100. Further, they were treated with phosphate buffed saline (PBS) containing 3% bovine serum albumin, incubated with anti-NFATc1 antibody, followed by incubation with an Alexa Fluor-488 conjugated secondary antibody (Invitrogen, Carlsbad, CA, USA). The cells were then stained with rhodamine-conjugated phalloidin (Cytoskeleton, Denver, CO, USA) and DAPI (Santa Cruz Biotechnology, Santa Cruz, CA, USA) to visualize F-actin and nuclei. Fluorescent images were captured using a BX51 fluorescence microscope (Olympus, Tokyo, Japan).

### 4.7. Bone Resorption Pit Assay

BMMs were seeded on bone slices (IDS Nordic Bioscience, Herlev, Denmark) and cultured in osteoclastogenic media for three days. Further, they were incubated with or without DPHC (75 μg/mL) for two more days. After removing the cells, the bone slices were stained with hematoxylin, and the bone resorption area was examined using the i-Solution image analysis program (IMT i-Solution; Daejeon, Korea).

### 4.8. Statistical Analysis

All experiments were performed thrice; results are presented as mean ± standard deviation. Statistical differences were evaluated using the two-tailed Student’s *t*-test or one-way analysis of variance (ANOVA) with Tukey’s multiple comparison *post-hoc* test; * *p* < 0.05 or ** *p* < 0.01 were considered significant.
